# New Molecular Markers Involved in Regulation of Ovarian Granulosa Cell Morphogenesis, Development and Differentiation during Short-Term Primary In Vitro Culture—Transcriptomic and Histochemical Study Based on Ovaries and Individual Separated Follicles

**DOI:** 10.3390/ijms20163966

**Published:** 2019-08-15

**Authors:** Magdalena Kulus, Patrycja Sujka-Kordowska, Aneta Konwerska, Piotr Celichowski, Wiesława Kranc, Jakub Kulus, Hanna Piotrowska-Kempisty, Paweł Antosik, Dorota Bukowska, Dariusz Iżycki, Małgorzata Bruska, Maciej Zabel, Michał Nowicki, Bartosz Kempisty

**Affiliations:** 1 Veterinary Center, Nicolaus Copernicus University in Torun, 87-100 Toruń, Poland; 2 Department of Histology and Embryology, Poznan University of Medical Sciences, 61-701 Poznan, Poland; 3 Department of Anatomy, Poznan University of Medical Sciences, 61-701 Poznan, Poland; 4 Department of Toxicology, Poznan University of Medical Sciences, 61-701 Poznan, Poland; 5 Chair of Biotechnology, Department of Cancer Immunology, Poznan University of Medical Sciences, 61-701 Poznan, Poland; 6 Department of Histology and Embryology, Wroclaw Medical University, 50-367 Wroclaw, Poland; 7 Division of Anatomy and Histology, University of Zielona Gora, 65-417 Zielona Góra, Poland; 8 Department of Obstetrics and Gynecology, University Hospital and Masaryk University, 601 77 Brno, Czech Republic

**Keywords:** pig, granulosa, cells morphogenesis

## Abstract

Nowadays, science has a lot of knowledge about the physiology of ovarian processes, especially folliculogenesis, hormone production and ovulation. However, the molecular basis for these processes remains largely undiscovered. The cell layer surrounding the growing oocyte—granulosa cells—are characterized by high physiological capabilities (e.g., proliferation, differentiation) and potential for growth in primary cultures, which predisposes them for analysis in the context of possible application of their cultures in advanced methods of assisted reproduction. In this study, we have used standard molecular approaches to analyze markers of these processes in primarily in vitro cultured porcine granulosa, subjected to conditions usually applied to cultures of similar cells. The material for our research came from commercially slaughtered pigs. The cells were obtained by enzymatic digestion of tissues and in vitro culture in appropriate conditions. The obtained genetic material (RNA) was collected at specific time intervals (0 h—before culture; reference, 48, 98, 144 h) and then analyzed using expression microarrays. Genes that showed a fold change greater than |2| and an adjusted *p* value lower than 0.05 were described as differentially expressed. Three groups of genes: “Cell morphogenesis”, “cell differentiation” and “cell development” were analyzed. From 265 differently expressed genes that belong to chosen ontology groups we have selected *DAPL1*, *CXCL10*, *NEBL*, *IHH*, *TGFBR3*, *SCUBE1*, *DAB1*, *ITM2A*, *MCOLN3*, *IGF1* which are most downregulated and *PDPN*, *CAV1*, *TMOD1*, *TAGLN*, *IGFBP5*, *ITGB3*, *LAMB1*, *FN1*, *ITGA2*, *POSTN* genes whose expression is upregulated through the time of culture, on which we focused in downstream analysis. The results were also validated using RT-qPCR. The aim of our work was to conduct primary in vitro culture of granulosa cells, as well as to analyze the expression of gene groups in relation to the proliferation of follicular granulosa cells in the model of primary culture in real time. This knowledge should provide us with a molecular insight into the processes occurring during the in vitro cultures of porcine granulosa cells, serving as a basic molecular entry on the extent of the loss of their physiological properties, as well as gain of new, culture-specific traits.

## 1. Introduction

The female reproductive system is an extremely rich environment with many different cell types. The granulosa layer of the ovarian follicle plays a key role in the nutrition of the oocyte. The growing oocyte undergoes several biochemical and morphological changes. This process occurs simultaneously with folliculogenesis, starting during early fetal development [1]. Ovaries in pigs form on the 26th day of pregnancy and follicles appear after about 40 days [2]. The oocyte is initially surrounded by one layer of granulosa cells located on the basal membrane. They protect the oocyte and ensure the supply of nutrients [3]. During the formation phase of the antral follicle we distinguish granulosa cells (GCs) and theca cells (TCs). An important aspect is also the participation of granulosa cells in the formation cumulus cell layer. It has also been shown [4] that granulosa, theca, basement membrane and capillaries are involved in the transfer of small metabolites to oocytes.

Our previous experience has shown that porcine granulosa cells can be cultured in vitro in short—term culture [5]. It was proved that these cells show logarithmic growth in vitro and significant expression of connexin 43 and cyclin—dependent kinase 4 mRNAs. Also, through detailed analyses, we have shown the movement of these proteins between the nucleus and cytoplasm in the cultured cells. This indicates the likely contribution of these molecules to nuclear—cytoplasmic shuttling of signals during cumulus—oocyte communication. Granulosa cells can be a great source of potential knowledge about complex processes occurring in cells of significant physiological interaction devoid of their usual environment, the molecular mechanisms of which still remain undiscovered. An extremely important aspect is the communication between oocytes and granulosa cells, which, among other things, play a role in the synthesis and expression of many hormones responsible for the development of gametes, but also for the processes associated with the physiology of reproduction, which adds the possibility that granulosa cultures could someday be used in advanced assisted reproduction techniques [6].

In our current research we have used microarray assays, together with RT-qPCR validation, to analyze the expression patterns of genes involved in the morphogenesis, differentiation and cellular development. The “cell differentiation” ontological group, contains genes that participate in processes during which undifferentiated cells acquire specialization, both morphological and functional, occupying a specific location in the mature body. On the other hand, the process whose specific outcome is the progress of the cell in time (but does not include the stages associated with the assignment of the cell to a specific fate) are regulated by genes belonging to “cellular development” gene ontology (GO). Then, the “cell morphogenesis” ontology group describes the genes involved in the development process, in which the size or shape of the cell is generated and organized. The aim of our work was to conduct primary in vitro culture of granulosa cells, to analyze the expression of gene groups in relation to the proliferation of follicular granulosa cells in the model of primary culture in real time. This knowledge should provide us with a molecular insight into the processes occurring during the in vitro cultures of porcine granulosa cells, serving as a basic molecular entry on the extent of the loss of their physiological properties, as well as gain of new, culture-specific traits. 

The presented studies indicate not only the possibility of GCs proliferation in primary culture conditions in vitro, but also the expression of genes involved in differentiation-related processes, indicates a new potential of GCs devout of their physiological environment. Several authors conducted similar analyzes on human GCs indicating the potential of these cells to differentiate into other cell types in primary in vitro culture. Kossowka-Tomaszczuk et al. and Kranc et al. indicate such properties of human GCs maintained in long-term primary culture. The presented studies relate to porcine cells, suggesting that short-term culture also possibly induces differentiative potential of GCs. The presented research should be treated as the first stage basic entry, identifying individual genes involved in the processes of differentiation and proliferation. The results aim to serve as a basis for further proteomic studies, results of which can be compiled with those presented in this manuscript and possibly extrapolated to become meaningful in clinical situations. Confirmation of the stem-like potential of GCs and the possibility of their differentiation towards other types of cells may be used primarily in regenerative and reconstructive medicine, as well as approaches associated with assisted reproduction techniques. The main aim of the study was to identify the genes responsible for the proliferation and differentiation of GCs in short-term primary in vitro cultures.

## 2. Results

Whole transcriptome profiling by Affymetrix microarray allowed us to analyze the granulosa gene expression changes at 48, 96 and 144 h of in vitro culture. With the use of Affymetrix^®^ Porcine Gene 1.1 ST Array Strip we examined expression of 27,558 transcripts. Genes with fold change higher than abs (2) and with a corrected p-value lower than 0.05 were considered as differentially expressed. This set of genes consists of 3380 different transcripts, complete list of which can be found in the GEO database (ID: GSE134361). 

**Figure 1:**
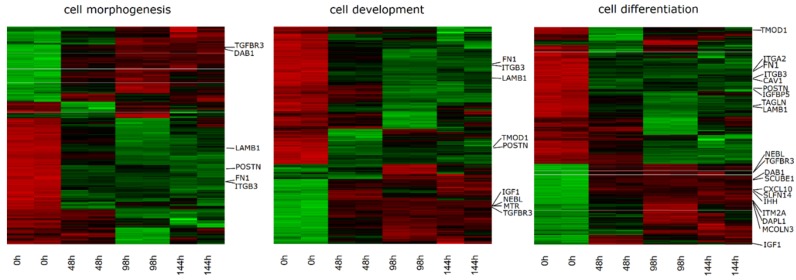
Heat map representation of differentially expressed genes belonging to the chosen “cell development”, “cell differentiation" and "cell morphogenesis” gene ontology (GO) BP terms. Arbitrary signal intensity acquired from microarray analysis is represented by colors (green, higher; red, lower expression). Log2 signal intensity values for any single gene were resized to Row z-score scale (from −2, the lowest expression to +2, the highest expression for a single gene). Due to the large number of genes on the heatmap, only the genes chosen for analysis in the manuscript were marked, with their names indicated.

Up and down regulated gene sets were subjected to the Database for Annotation, Visualization and Integrated Discovery (DAVID) search separately and only ones with an adj. *p* Value lower than 0.05 were selected. The DAVID software analysis showed that the differently expressed genes belonged to 344 GO BP Terms. In this paper we focused on the chosen “cell development”, “cell differentiation” and “cell morphogenesis” GO BP terms. These sets of genes were subjected to a hierarchical clusterization procedure and presented as heatmaps (Figure 1). There were 265 genes of differential expression in the gene ontologies of interest, from which 114 genes were downregulated and 151 were upregulated compared to the reference point. The gene symbols, fold changes in expression, Entrez gene IDs and corrected *p*-values of that genes are shown in Table 1.

**Figure 2:**
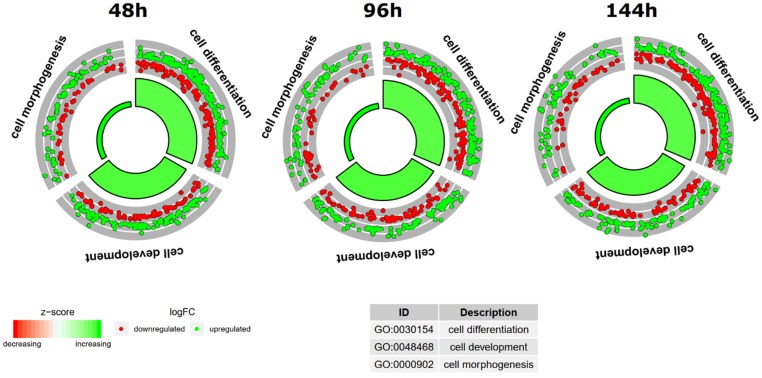
The circular visualization of the results of gene-annotation enrichment analysis. The outer circle shows a scatter plot for each term of the logFC of the assigned genes. Red circles display up-regulation and blue ones down-regulation. The inner circle is the representation of the z-score. The size and the color of the bar correspond to the value of z-score.

To further investigate the changes within the chosen GO BP terms, we measured the enrichment levels of each selected GO BPs. The enrichment levels were expressed as z-scores and presented as circular visualizations (Figure 2). As can be seen in the Figure, all three ontological groups contain a larger proportion of genes of increased expression (green color of the inner ring), with “cell differentiation” and “cell development” containing the biggest number of genes. 

**Figure 3:**
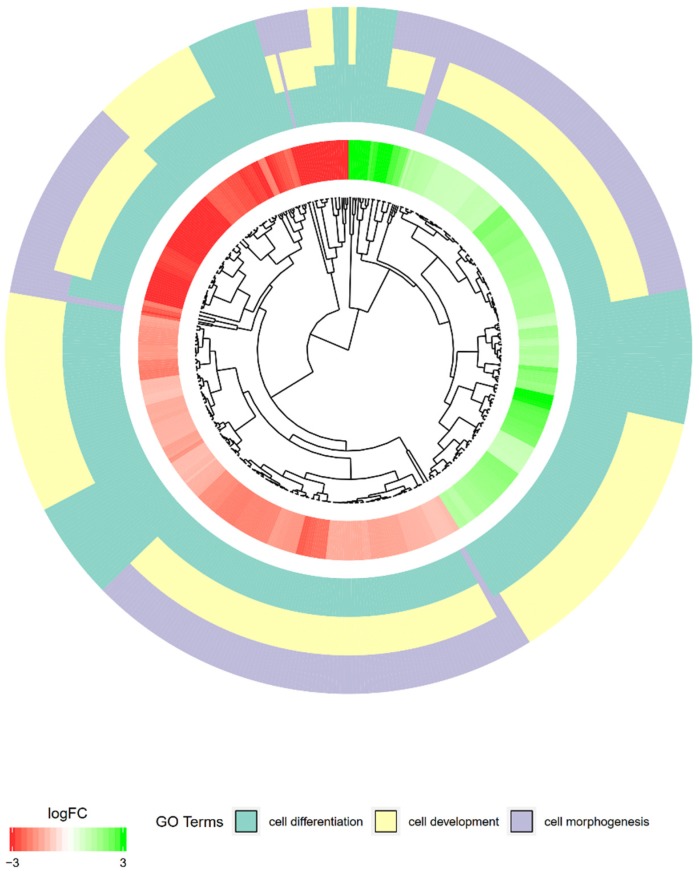
The representation of hierarchical clusterization, fold change and assignment of differently expressed genes that belongs to chosen “cell development”, “cell differentiation” and “cell morphogenesis” GO BP terms. Genes are grouped together based on their expression patterns, with the clusterization pattern represented by dendrogram inside the circle. The middle ring represents the logarithm of gene expression fold change of studied genes. The outer ring represents the terms assigned to the genes.

To better understand the interaction between chosen GO BP terms we performed the hierarchical clusterization of the gene expression profiles. The resulting dendrogram was combined with fold changes (FC) of studied gene expression and gene assignment to studied terms. The results were presented in Figure 3. Most of the genes of altered expression that belong to “cell morphogenesis” GO are also present in the other gene ontologies of interest. Unique genes of differential expression that are not a part of other gene ontologies, appear mostly in the “cell development” group, with minor fractions characteristic for “cell morphogenesis”. “Cell differentiation” did not contain any differentially expressed genes unique only for that group. 

Among 265 differently expressed genes that belongs to GO BP terms of interest we have chosen 10 with most upregulated and 10 with most downregulated expression levels, on which we focused during downstream analysis.

**Figure 4:**
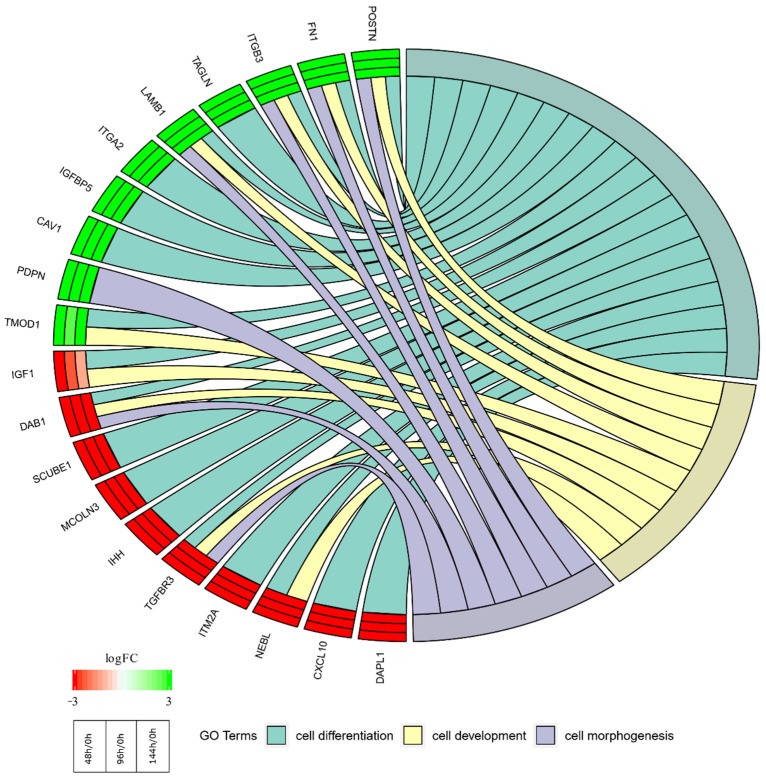
The representation of the mutual relationship between 10 most upregulated and 10 most downregulated genes that belongs to “cell development”, “cell differentiation” and “cell morphogenesis” GO BP terms. The ribbons indicate which gene belongs to which categories. The genes were sorted by logFC from most to least changed gene, with the most upregulated genes presented topmost and the most downregulated genes located at the bottom of the figure. The tips of the ribbons present the direction of the change (upregulation-green; downregulation-red), with each of their parts showing change in particular culture period (innermost—48 h/0 h; middle—96 h/0 h; outermost—144 h/0 h).

**Figure 5:**
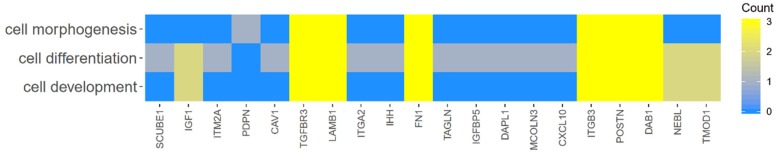
Heatmap showing the gene occurrence between 10 most upregulated and 10 most downregulated genes that belongs to “cell development”, “cell differentiation” and “cell morphogenesis” GO BP terms.

In the gene ontology database, genes that form one particular GO group can also belong to other different GO term categories. For this reason, we explore the gene intersections between the selected GO BP terms. The relation between those GO BP terms was presented as a circle plot (Figure 4) as well as the heatmap (Figure 5). As can be seen on the figures, most (11/20) genes of the most altered expression is characteristic uniquely for the “cell differentiation” group, with the remaining seven being members of all three GOs of interest. 

**Figure 6:**
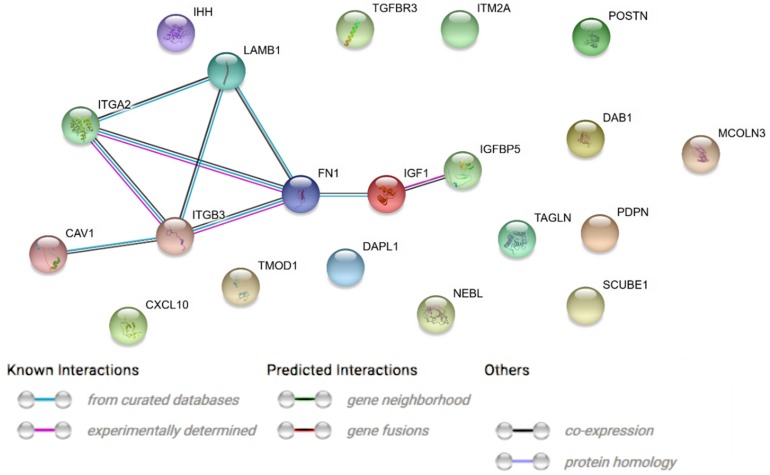
STRING-generated interaction network among differentially expressed genes belonging to the between 10 most upregulated and 10 most downregulated genes that belongs to “cell development”, “cell differentiation” and “cell morphogenesis” GO BP terms. The intensity of the edges reflects the strength of interaction score.

A STRING interaction network was generated among chosen differentially expressed genes belonging to each of the selected GO BP terms [7]. Using such a prediction method provided us with a molecular interaction network formed between protein products of studied genes (Figure 6). According to the STRING database, the biggest amount of confirmed interactions can be observed between *POSTN*, *FN1*, *ITGB3*, *ITGA2* and *DAB1* genes. Some interactions between *IGF1*, *IGFBP5*, *CAV1* and *LAMB1* genes were also observed. 

**Figure 7:**
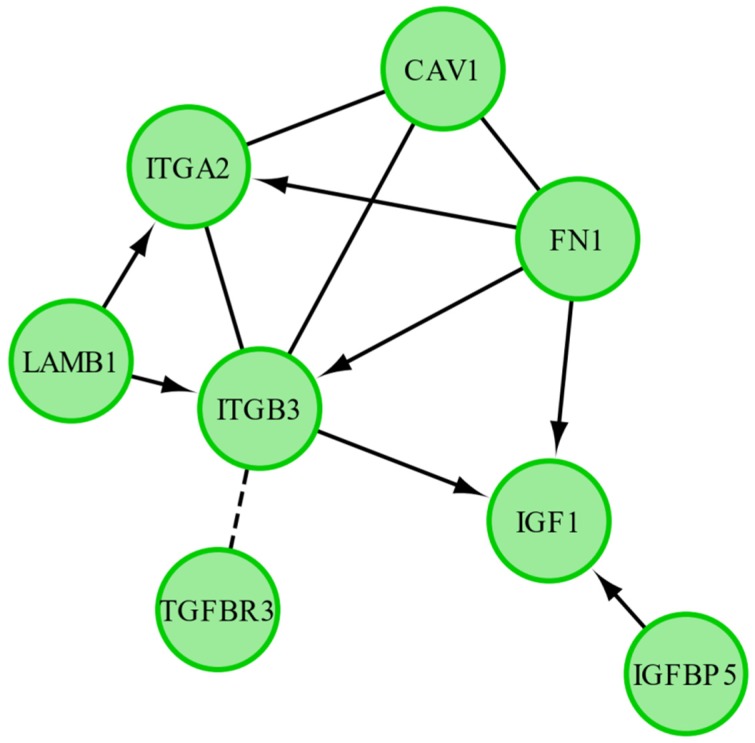
Functional interaction (FI) between 10 most upregulated and 10 most downregulated genes that belong to “cell development”, “cell differentiation” and “cell morphogenesis” GO BP terms. In the figure “→” stands for activating/catalyzing, “—” stands for FIs extracted from complexes or inputs, and “---” stands for predicted FIs.

Finally, we have investigated the functional interactions between chosen genes with the REACTOME FIViz app to Cytoscape 3.6.0 software. The results are shown in Figure 7.

The results of this figure present interactions between eight out of 20 genes of interest. *FN1* catalyses the expression of three genes (*ITGA2*, *ITGB3*, *IGF1*), *LAMB1* catalyses the expression of two genes (*ITGA*, *ITGB3*). Finally, the expression of IGF1 is catalysed by three genes (*IGFBP5*, *FN1*, *ITGB3*). 

**Figure 8:**
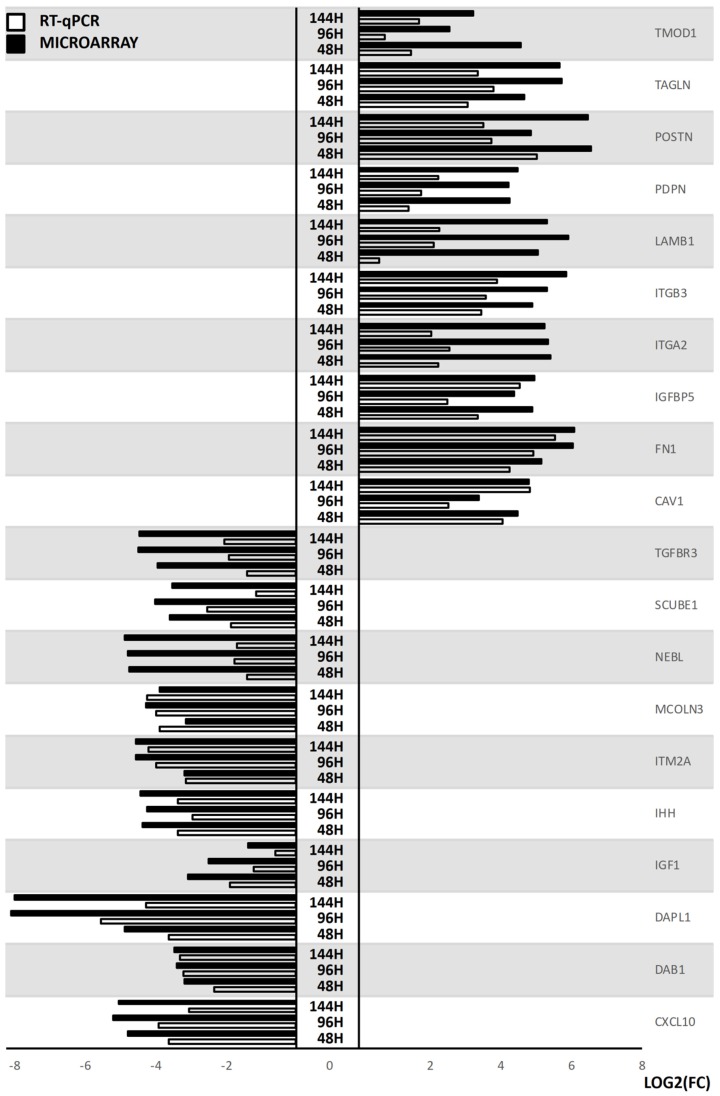
The results of RT-qPCR validation of the analysed genes, presented in the form of bar graph. All of the changes in expression were deemed significant at *p* < 0.05 after adjustment for multiple comparisons.

The results of the microarray analysis were validated with the RT-qPCR methods. The obtained values were compared between both approaches and are presented as a bar graph (Figure 8).

Overall, the direction changes in the expression of genes of interest were validated in every example. However, their scale often varies highly between the methods. This fact is explainable, as these methods are different in sensitivity and specificity, which further supports the need for quantitative validation of microarray results. 

**Figure 9:**
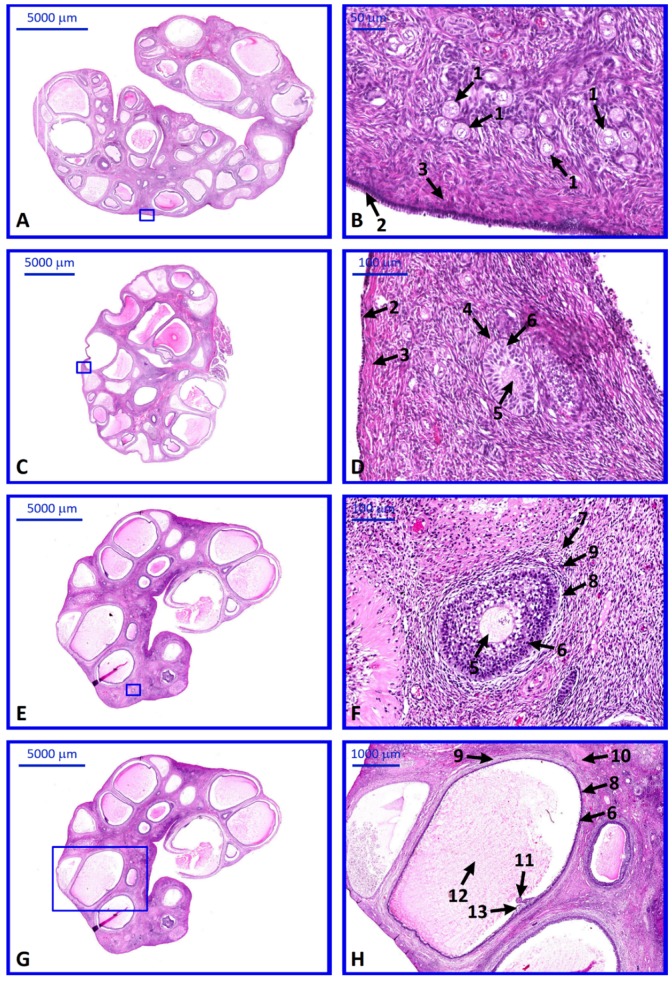
H and E stained ovaries (**A**,**C**,**E**,**G**) with follicles in particular stages of development (**B**,**D**,**F**,**H**). **B**,**D**,**F**,**H** represent selected areas of **A**,**C**,**E**,**G** observed in higher magnification (with blue square indicating the magnified region). Arrows: 1—primordial follicles, 2—germinal epithelium, 3—tunica albuginea, 4—primary follicle, 5—oocyte, 6—granulosa cells, 7—secondary follicle, 8—theca interna, 9—theca externa, 10—mature follicle, 11—oocyte with corona radiata, 12—antrum, 13—cumulus oophorus.

Histochemical studies revealed the proper morphology of collected ovaries stained with H and E. Ovarian follicles in every stage of development were observed (Figure 9). 

**Figure 10:**
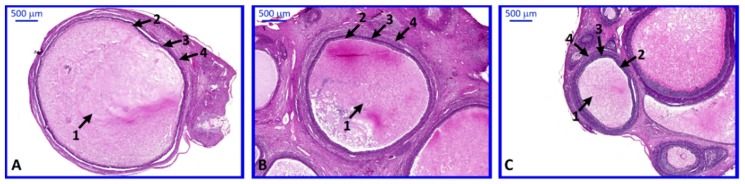
Individual isolated follicles stained with routine H and E method. Follicles varied in size; we can observe large (>5 mm), medium (3–5 mm) and small follicles (<3 mm). Medium and small follicles were collected in groups containing more than one follicle. (**A**)—large follicle, (**B**)—medium follicle, (**C**)—small follicle. Arrows: 1—antrum, 2—granulose cells, 3—theca interna, 4—theca externa.

Individual isolated follicles varied in size. Large (>5 mm), medium (3–5 mm) and small (<3 mm) follicles were observed (Figure 10).

## 3. Discussion

Granulosa cells are known to be closely related to mammalian oocytes. They surround them from the very beginning, and still in fetal life, support their development, maturation, and formation of ovarian follicles [8]. They are associated with the functioning of the reproductive system, as well as the maintenance of pregnancy through participation in the synthesis of steroid hormones [9]. After ovulation, when the follicle ruptures, the granulosa cells fill the formed gap and transform into lutein cells, whose main purpose is to produce progesterone and maintain pregnancy. The resulting structure is called the corpus luteum. The physiology of these processes is well known, but the deep molecular mechanisms and gene interactions of granulosa cells are still insufficiently described [10]. Extremely dynamic changes in the area of the mammalian mature ovary require adequate supply of blood vessels. Particularly dynamic processes related to angiogenesis occur in the corpus luteum. In a short time, a network of blood vessels is created, allowing cells with hormonal activity to obtain oxygen, nutrients, as well as hormone precursors necessary for the synthesis and release of progesterone in the early stages of pregnancy [11]. In modern human medicine and advanced procedures of assisted reproduction techniques in farm animals, understanding of all the mechanisms influencing the increase of reproductive efficiency seems to be of key importance. In addition, recent studies prove that ovarian granulosa cells can gain and/or exhibit programmed stem cell properties during in vitro culture [12,13,14]. 

Using modern research methods, we have the opportunity to learn more about the molecular basis of functioning of such microenvironments as ovarian follicles. Pigs, as animals physiologically similar to humans, are a commonly used models in medical research. Only through detailed analysis will it be possible to get deeper into the background of some clinical diseases and perhaps to get closer to the paths of their solution. Research on cells of the reproductive system can lead to new strategies for increasing the reproductive potential of animals and treating infertility in humans.

In our present research, we have measured the gene expression changes in three ontological groups (“cell morphogenesis”, “cell differentiation”, “cell development”), using the microarray approach. Our experiments were based on the successful primary short-term culture of granulosa cells (GCs) obtained from ovaries of pigs killed in a slaughterhouse. The obtained cells were subjected to short term in vitro culture, up to 144 h. RNA material was isolated from the cells before the primary culture (0 hour) and then after 48, 96 and 144 h of its course. We analyzed 20 genes that showed the biggest changes in expression. From 265 differentially expressed genes belonging to the GOs of interest, we have selected *DAPL1*, *CXCL10*, *NEBL*, *IHH*, *TGFBR3*, *SCUBE1*, *DAB1*, *ITM2A*, *MCOLN3*, *IGF1*, showing the biggest upregulation, as well as *PDPN*, *CAV1*, *TMOD1*, *TAGLN*, *IGFBP5*, *ITGB3*, *LAMB1*, *FN1*, *ITGA2*, *POSTN* genes whose expression was downregulated through the time of culture. All of the abovementioned genes were focused on during downstream analysis. Six of these genes belonged to all three groups (*TGFBR3*, *DAB1*, *ITGB3*, *LAMB1*, *FN1*, *POSTN*), and three others to two groups (*NEBL*, *IGF1*, *TMOD1*—cell differentiation and cell development). In all genes belonging to the three ontological groups of interest, a similar, uniform pattern of expression was observed. The 0-h expression analysis, used as a reference point, exhibited the lowest levels of expression. A significant increase in expression was described in the subsequent culture hours (48, 98, 144 h), which concerned almost all genes from the three groups. The value of 0 h, the starting point of cultures, was assumed as a point of reference in this study. This method served to fulfil the aim of comparing the gene expression patters in different timepoints of in vitro cultures. Hence, it was assumed that 0h relatively well reflects the physiological gene expression of granulosa cells.

The most down-regulated gene was *DAPL1* (death associated protein—like 1), belonging to an ontological group of “cell differentiation”. Its role is described in the early stages of epithelial differentiation, but also in the processes of apoptosis. Significant increases in *DAPL1* gene expression was observed in the study on uterine inflammation in dairy cows, analyzing levels of its transcripts in endometrium [15]. The downregulation of this gene’s expression in subsequent cultures stages suggests a change in the properties of granulosa cells during primary in vitro culture. A similar pattern of expression is exhibited by the 9 genes described below. The next gene, *CXCL10* (*C-X-C* motif chemokine ligand 10) also belonging to the “cell differentiation" group is also known as interferon–gamma inducible protein-10 (IP-10). The *CXCL10* gene was described in pigs by Liu and Xiong, where it was proven to be highly expressed in muscle and weakly expressed in fat and kidneys [16]. The *CXCL10* gene codes a cytokine associated with inflammatory processes, such as activation of immune cells, differentiation, but also apoptosis or cell growth regulation. Another gene that shows significant down-regulation in our in vitro studies of cultured GCs in relation to the reference point is *NEBL*. NEBL (nebulette) is a protein that binds to actin and plays an important role in Z-disk assembly. Mainly found in the heart muscle, where it combines sarcomeric actin with desmin fibers in sarcomers [17]. We observed a significant decrease in the expression of this gene in GCs’ primary culture. *Indian hedgehog protein* (*IHH*) is an important gene encoding an intracellular signaling protein that plays a role in cell development. A special role is attributed to *IHH* in the process of ossification and mesenchymal cell proliferation [18]. This gene belongs to the “cell differentiation” group and was definitely upregulated. *TGFBR3* (*transforming growth factor beta receptor 3*) is an important signal point for cell differentiation and development, also in such an important process as the formation of coronary vessels in the heart [19]. *TGFBR3* contributes to the response to the FSH hormone signals. The decrease in these gene’s expression in subsequent days of culture suggests a change in properties of granulosa cells. Hence, our future studies could Focus on the secretive properties of GCs in in vitro culture conditions. Furthermore, *TGFBR3* also plays a role in the epithelial to mesenchymal transition. It was detected, among others, in the porcine uterus [20]. *SCUBE1* (*signal peptide CUB domain and EGF like domain containing 1*) is closely related to endothelial cells (differentiation) and vascular system. It occurs in highly vascularized tissues. Its expression also manifests a strong connection with the cell surface [21]. This gene, belonging to “cell differentiation” GO, showed down-regulation during our culture. Subsequently, *DAB1* (*DAB protein adaptor 1*) is a gene encoding adaptive protein necessary for intracellular Reelin signal transmission. This in turn controls the migration and differentiation of postmitotic neurons in the brain development process [22]. We have determined that this gene belongs to all three ontological groups, which reflects its role in morphogenesis, differentiation and cellular development. We observed a significant downregulation of *DAB1*, which probably indicates the loss of physiological properties of the aforementioned processes in in vitro cultured porcine granulosa cells. It is possible that this gene may become a marker of neuronal differentiation of granulosa cells, which requires further research. Considering the aim of our research, the expression of the abovementioned four genes is particularly important. Their high expression at 0h suggests that granulosa cells exhibit mesenchymal stem cell like potential in physiological conditions. The next gene, the *ITM2A* (*integral membrane of 2A protein*) encodes a porcine protein that is highly homologous to the integrated 2A (ITM2A) membrane protein of humans and mice. This gene is described in fat and spleen, in the lungs, as well as in the muscles, liver, small intestine, large intestine and kidneys [23]. In our studies, we have shown a significantly reduced expression of this gene, which only belongs to the “cell differentiation” gene ontology. Another gene of decreased expression from the “cell differentiation” GO is *MCOLN3* (mucolipin 3), which was found in various pig tissues, also in the ovary [24]. The important role of *MCOLN3* is described in the regulation of Ca^2+^ homeostasis in the endosomal pathway, as well as in melanosomal transduction and hair cell maturation, as demonstrated by mouse studies [25]. The last down-regulated gene was *IGF1* (*insulin like growth factor 1*), belonging to the “cell differentiation” and “cell development” groups. This gene was analyzed in studies on ovarian follicle development in cattle and pigs in order to compare single and multiovulatory animals. These experiments showed that IGF1 was one of the factors limiting or promoting multiple ovulation [26]. Therefore, it can be assumed that high expression in 0h of this gene in the in vitro culture of granulosa cells may testify to the ability of these cells to regulate the ovulation processes in physiological condition. *IGFBP5* (*insulin like growth factor binding protein 5*), which has shown up–regulation, is not correlated with the IGF1 gene. The presence of these genes’ expression was shown in the ovary and other organs of pigs [24]. The only gene that belongs exclusively to the “cell morphogenesis” group is *PDPN*, which may predispose it to becoming a marker of in vitro GC culture morphogenesis. *PDPN* (*podoplanin*) is a protein–coding gene that is quite widespread in porcine lungs, ovaries and other tissues. In our research it was up-regulated during the in vitro culture of GCs. The gene was also described in studies of pancreatic cancer, where fibroblasts expressed increased expression of *PDPN*. Podoplanin, a transmembrane glycoprotein, is selectively expressed by lymphatic endothelial cells [27]. It is possible that its presence in culture is related to cell differentiation in the vascular direction, because it was strongly associated with neoplastic angiogenesis. The *TMOD1* gene (*tropomodulin 1*) was found to belong to two ontological groups (“cell differentiation” and “cell development”). The protein coded by this gene plays an important role in the physiology of muscles, especially skeletal muscles, and its expression was mainly noted in the psoas major. An increase in the expression of these gene in subsequent stages of the culture suggests that granulosa cells gain new properties when cultured in vitro. The *TAGLN* (*transgelin*) gene is associated with the differentiation of epithelium in human intestine cell line cultures [28]. In GC, in vitro primary culture, this gene was up–regulated and belonged to the "cell differentiation" group. *FN1* (*fibronectin 1*) represents all three ontological groups, and its expression was recorded to be upregulated. Significant upregulation of the *FN1* gene was observed in an experiment on the primary culture of pig buccal mucosa cells in vitro. It was observed that increased expression of this gene, among other factors, was accompanied by cellular proliferation [29]. Fibronectin is a protein commonly found in extracellular matrix of blood vessels but also in dissolved form in blood [30]. Increased expression suggests that fibronectin is also present in the GC extracellular matrix. It activates ITGB3 (integrin subunit beta 3) proteins, which also showed upregulation in our experiment and belong to the same three ontological groups. By analyzing the gene correlation (STRING) we also observed the co-expression of proteins coded by these two genes. *ITGB3* also shows co–expression with downregulated *IGF1* gene and two of the analyzed upregulated genes: *ITGA2* and *LAMB1*. The *LAMB1* gene (*laminin subunit beta 1*) mediates the migration and organization of cells to tissues during embryonic development through interaction with other extracellular matrix components. It also plays a role in embryo implantation, as indicated by a mice study [31]. Laminin protein plays a role in the regulation of tissue proliferation, differentiation and repair [32]. In our study, this gene showed upregulation and belonged to all three analyzed ontological groups. Notable expression of this gene suggests that in vitro cultured GCs might gain new properties, including the potential to differentiate into other cell types. Another gene that plays a role in the early stages of embryogenesis [33] is *ITGA2* (*integrin subunit alpha 2*). Its expression was recorded in porcine kidneys, ovaries and other tissues. The correlation with *FN1* and *ITGB3* genes was also found for the *CAV1* gene (caveolin 1). This gene belongs only to the "cell differentiation" group and, like the two genes mentioned above, it was upregulated. The proteins encoded by these genes can form interactive complexes participate in cell differentiation during angiogenesis [34]. The *FN1* and *POSTN* genes are strongly correlated, showing co-expression and belonging to all three ontological groups. The *POSTN* gene (*periostin*) is responsible for cellular adhesion and bone regeneration [35]. In our research, this gene indicated the strongest up–regulation. Using STRING-generated interaction network, we have observed that 11 of the 20 analyzed genes did not show direct interactions with other genes. However, indirect interactions cannot be excluded, either through genes not detected in this study or those that have not shown significant changes in expression.

Analyzing the above results, it should be remembered that the change in the expression of individual genes may result not only from changes in nucleotide sequences but also from epigenetic changes occurring during the change of environment from physiological to ex vivo. Many factors, including age and obesity, have an effect on reproduction and epigenetics of germ cells [36]. Studies conducted on human cumulus cells suggest that the quality of oocytes and implantation of embryos may depend on whether the woman is overweight or not. Much less mature oocytes and inferior embryos were obtained from obese women than from women without obesity [37]. 

To summarize, our research showed expression of several genes of porcine ovarian granulosa cells, which manifested during short-term primary in vitro culture. Three ontological groups of genes were distinguished: “Cell morphogenesis”, “cell development” and “cell differentiation”. The observed gene expression data may indicate advanced molecular processes connected with proliferation of granulosa cells in in vitro culture. The vast majority (19 out of 20) of the described genes belonged to the “cell differentiation” group, while the “cell morphogenesis” group had the least representatives (7 genes). Some of these genes play a role in angiogenesis, which occurs abundantly in the ovary. Significant expression of genes from the described groups may reflect the ability of GCs to rapidly multiply and differentiate in the corpus luteum. However, it can also suggest the appearance of novel differentiation properties achieved during the course of in vitro culture. Our research is intended to provide further clarification to the molecular bases of processes occurring during the short-term in vitro culture of granulosa cells. The results partly support the thesis that some cells devoid of their physiological environment lose their original properties, instead gaining potential for differentiation into other cell types. However, it needs to be noted that this manuscript describes an entry level study. While there is a possibility that the results can be extrapolated into knowledge applicable in clinical situations, they need further validation on the protein level, followed by additional studies conducted in in vivo or simulated clinical conditions. Hence, these outcomes could serve as an entry-level molecular reference for further studies of stem-like properties of ovarian granulosa cells, as well as their potential application in the processes of assisted reproduction of animals and humans. 

## 4. Materials and Methods

### 4.1. Animals

A total of 43 crossbred Landrace gilts with a median age of 170 days and weight of 98 kg were used in this study. All animals were housed under identical conditions. Pigs acquire sexual maturity between 4-6 months of age, and therefore the material obtained came from young, sexually mature, gilts ready for reproduction. All of the specimen were slaughtered in the anestrous stage of the estrous cycle. As the ovaries were obtained from animals of relatively equal age, the collected samples exhibited high homogeneity. The experiment was approved by Poznan University of Life Sciences Bioethical Committee (Resolution 32/2012, approved 1 June 2012). 

### 4.2. Collection of Porcine Ovaries and In Vitro Culture of Granulosa Cells

Ovaries (*n* = 86) and reproductive tracts were recovered at slaughter and transported to the laboratory at 38 °C in 0.9% NaCl within 30 min. The ovaries of each animal were placed in PBS supplemented with fetal bovine serum (FBS; Sigma-Aldrich Co., St. Louis, MO, USA). Thereafter, single preovulatory large follicles, with an estimated diameter greater than 5 mm (*n* = 300), were opened into a sterile Petri dish by puncturing with a 5 mL syringe and 20-G needle. The cumulus-oocyte complexes (COCs) and follicular fluid (FF) were recovered. The follicular fluid was the used to isolate GCs, whereas the COCs were discarded. The method employed, as well as the choice of material was chosen to most closely reflect the usual approaches used in assisted reproduction techniques, as this study could serve as a reference for future research aiming to improve the processes of in vitro fertilization and assisted reproduction of animals and possibly humans. 

The culture medium consisted of Dulbecco’s Modified Eagle’s Medium (DMEM, Sigma-Aldrich), 2% fetal calf serum (FCS; increased concentration required to maintain adequate cell viability in culture) (PAA, Linz, Austria), 10 mg/mL ascorbic acid (Sigma-Aldrich), 0.05 μM dexamethasone (Sigma-Aldrich), 200 mM L-glutamine (Invitrogen, Carlsbad, CA, USA), 10 mg/ml gentamycin (Invitrogen), 10,000 units/mL penicillin and 10,000 μg/mL streptomycin (Invitrogen). The cells were cultivated at 38.5 °C under aerobic conditions (5% CO_2_). Once the adherent cells were more than 80% confluent, they were detached with 0.05% trypsin-EDTA (Invitrogen) for 3 min and counted using a Z2 counter or cell viability analyzer (Vi-Cell XR 2.03; both Beckman Coulter, Brea, CA, USA). For our experiments, 3 × 10^6^ cells per dish were used for the culture, with the number achieved by adequate pooling of the material obtained from the punctured follicles into samples of comparable cell number. The cell samples were collected in 4 time intervals, representing different stages of short-term in vitro culture: 0 h—serving as an ex vivo reference; 48 h—representing initial in vitro associated changes in culture; 96 h—an assumed “point of loss” of most of the cell’s physiological properties; 144 h—the end point of short-term culture.

### 4.3. Histological Analysis

For the histochemical study ovaries and single follicles from 3 animals were collected and immediately fixed in Bouin’s solution for 48 h. Due to technical limitations, follicles smaller than 5 mm were pooled into groups usually containing from 3 to 5 follicles. Subsequently, tissues were dehydrated and embedded in paraffin blocks. Then, they were cut into 4 µm thick sections with a semi-automatic rotary microtome (Leica RM 2145, Leica Microsystems, Nussloch, Germany). Both ovaries and single follicles were stained with a routine hematoxylin and eosin (H and E) staining method, following the steps of: Deparafinization and rehydration, H and E staining, and dehydration. Histological sections were observed and evaluated under light microscope. Selected pictures were taken with high-resolution scanning technique and Olympus BX61VS microscope scanner (Olympus, Tokyo, Japan).

### 4.4. Microarray Expression Analysis and Statistics

The Affymetrix procedure was previously described by Trejter et al. [38] and was used in our previous works regarding porcine oviductal cells [39,40,41] and porcine oocytes [42,43,44]. The cDNA was reverse transcribed from the total RNA (100 ng) (Ambion^®^ WT Expression Kit). Obtained cDNA was biotin labeled and fragmented using Affymetrix GeneChip^®^ WT Terminal Labeling and Hybridization Kit (Affymetrix, Santa Clara, CA, USA). Biotin-labeled fragments of cDNA (5.5 μg) were hybridized to Affymetrix^®^ Porcine Gene 1.1 ST Array Strip (48 °C/20 h). Then, the microarrays were washed and stained according to the technical protocol of the Affymetrix GeneAtlas Fluidics Station. Subsequently, the array strips were scanned by the Imaging Station of GeneAtlas System. The preliminary analysis of the scanned chips was performed using Affymetrix GeneAtlas^TM^ Operating Software. The quality of gene expression data was checked according to quality control criteria provided by the software. Obtained CEL files were imported into the downstream data analysis software. All of the presented analyses and graphs were performed using Bioconductor and the R programming language. Each CEL file was merged with a description file. In order to correct background, normalize and summarize results, we have used the robust multiarray averaging (RMA) algorithm.

Statistical significance of analyzed genes was performed by moderated *t*-statistics from the empirical Bayes method. The obtained *p*-value was corrected for multiple comparisons using the Benjamini and Hochberg’s false discovery rate. The selection of genes of significantly changed expression was based on a *p*-value beneath 0.05 and expression fold-change higher than 2. Differentially expressed gene lists (separate for up and downregulated genes) were uploaded into DAVID software (Database for Annotation, Visualization and Integrated Discovery) [45], where differentially expressed genes belonging to “cell development”, “cell differentiation” and “cell morphogenesis” were obtained. Expression data of these genes was subjected to hierarchical clusterization procedure and presented as a heatmap graph.

### 4.5. Real-Time Quantitative Polymerase Chain Reaction (RT-qPCR) Analysis

Total RNA was isolated from GCs before and after in vitro culture using RNeasy mini column from Qiagen GmbH (Hilden, Germany). The RNA samples were resuspended in 20 µL of RNase-free water and stored in liquid nitrogen. The samples were treated with DNase I and reverse-transcribed (RT) into cDNA. RT-qPCR was conducted in a LightCycler real-time PCR detection system (Roche Diagnostics GmbH, Mannheim, Germany) using SYBR^®^ Green I as a detection dye. Target cDNA was quantified using the relative quantification method. The relative abundance of the analyzed transcripts in each sample was standardized to the internal standards. For amplification, 1 µL of cDNA solution was added to 9 µL of QuantiTect^®^ SYBR^®^ Green PCR (Master Mix Qiagen GmbH, Hilden, Germany) and primers (Table 2). One RNA sample of each preparation was processed without the RT-reaction to provide a negative control for subsequent PCR.

To quantify the specific genes expressed in the GCs, the expression levels of specific mRNAs in each sample were calculated relative to *PBGD* and *ACTB*. To ensure the integrity of these results, the additional housekeeping gene, *18S rRNA*, was used as an internal standard to demonstrate that *PBGD* and *ACTB* mRNAs were not differentially regulated in GC groups. *18S rRNA* has been identified as an appropriate housekeeping gene for use in quantitative PCR studies. Again, the statistical significance of analyzed genes was performed by moderated t-statistics from the empirical Bayes method. The obtained *p*-value was corrected for multiple comparisons using the Benjamini and Hochberg’s false discovery rate. 

## Figures and Tables

**Table 1:** Fold changes, adjusted *p* values and ENTREZ gene ID of the 20 chosen differentially expressed genes belonging to the “cell morphogenesis”, “cell development” and “cell differentiation gene ontology (GO) terms.

Gene	FC 48 h/0 h	FC 96 h/0 h	FC 14 h4/0 h	*p* Value 48 h/0 h	*p* Value 96 h/0 h	*p* Value 144 h/0 h	Entrez ID
*CXCL10*	0.036689	0.027744	0.030979	3.47 × 10^6^	1.07 × 10^6^	1.53 × 10^6^	3627
*DAB1*	0.112175	0.096043	0.092698	3.33 × 10^6^	1.07 × 10^6^	1.26 × 10^6^	1600
*DAPL1*	0.03486	0.003772	0.004046	1.00 × 10^6^	9.14 × 10^8^	9.57 × 10^8^	92196
*IGF1*	0.119841	0.180781	0.391369	9.96 × 10^5^	0.000172	0.006284	3479
*IHH*	0.049771	0.0538	0.047738	2.16 × 10^6^	1.06 × 10^6^	1.09 × 10^6^	3549
*ITM2A*	0.11303	0.043537	0.042942	7.74 × 10^6^	7.05 × 10^7^	8.20 × 10^7^	9452
*MCOLN3*	0.114236	0.052489	0.068606	9.09 × 10^6^	1.06 × 10^6^	1.98 × 10^6^	55283
*NEBL*	0.037428	0.037303	0.034729	9.56 × 10^7^	3.68 × 10^7^	4.09 × 10^7^	10529
*SCUBE1*	0.084451	0.063451	0.087118	4.12 × 10^6^	1.09 × 10^6^	2.53 × 10^6^	80274
*TGFBR3*	0.065878	0.045288	0.04622	4.56 × 10^7^	1.51 × 10^7^	1.50 × 10^7^	7049
*CAV1*	22.31557	10.4332	28.12126	4.28 × 10^7^	4.59 × 10^7^	1.50 × 10^7^	857
*FN1*	35.37896	66.1651	68.74185	3.59 × 10^7^	9.92 × 10^8^	9.57 × 10^8^	2335
*IGFBP5*	29.89599	20.97384	31.2743	4.42 × 10^7^	3.09 × 10^7^	2.28 × 10^7^	3488
*ITGA2*	42.67896	41.0013	38.26346	3.52 × 10^7^	1.21 × 10^7^	1.26 × 10^7^	3673
*ITGB3*	29.91255	39.85199	57.68212	3.52 × 10^7^	1.21 × 10^7^	9.57 × 10^8^	3690
*LAMB1*	33.30078	60.54652	39.6371	3.52 × 10^7^	9.14 × 10^8^	9.57 × 10^8^	3912
*HPDPN*	18.98888	18.65575	22.39775	2.14 × 10^5^	1.11 × 10^5^	1.07 × 10^5^	10630
*POSTN*	95.22897	29.49005	88.94606	3.52 × 10^7^	1.80 × 10^7^	9.57 × 10^8^	10631
*TAGLN*	25.56666	53.77139	50.96687	3.52 × 10^7^	9.14 × 10^8^	9.57 × 10^8^	6876
*TMOD1*	23.71613	5.946509	9.468941	1.00 × 10^6^	6.63 × 10^6^	2.71 × 10^6^	7111

**Table 2:** Oligonucleotide sequences of primers used for RT-qPCR analysis.

Gene	Gene ID	Primer Sequence (5′–3′)	Product Size (bp)
*CXCL10*	494019	CTCCTGAAAGGCCCATCATA GCACATGGGATAGAGGAGGA	193
*DAB1*	100037307	TACGTTTGTGGGAAGGAAGG CTTCCTTCTTTTGGCTGGTG	202
*DAPL1*	100157537	CCTGCTCTGGAGAAGGTCAC GGGCCTAAGGAAAGTTTTGG	151
*IGF1*	47523587	TTCTACTTGGCCCTGTGCTT CTCCAGCCTCCTCAGATCAC	222
*IHH*	397174	CTCCACTGCCCTCTCAGAAC AGCTCGCAGCTGTGTCACTA	182
*ITM2A*	595131	TCTCGTAGGCCTTTCCTTCA AGGCAGGAAGTAGGGCTCTC	163
*MCOLN3*	100625693	TCCGAGTGCCTTTTCTCACT CGGATATAAACGTGCGGAGT	238
*NEBL*	100522395	CAAACCCTTCAAGGCTACCA CTGAGAACACGCTTCCATCA	177
*SCUBE1*	100524621	AATCCAATGAAGCCAACAGC AGGGCCTTGATCAGCTTCTT	160
*TGFBR3*	397512	TTTGTTTTAGCTGGGGGTTG TGGCCACAGGGATTTTTAAG	177
*CAV1*	404693	TAGGTCAGCAGCCTCCCTAA CTGGTGAGAGGCAGGAAAAG	243
*FN1*	397620	TGAGCCTGAAGAGACCTGCT CAGCTCCAATGCAGGTACAG	113
*IGFBP5*	397182	TGGGGGTTTGTTTCTCTGAC TTCTGGCAGGTAGAGCAGGT	181
*ITGA2*	397483	CATGCCAGATCCCTTCATCT CGCTTAAGGCTTGGAAACTG	153
*ITGB3*	397063	GGCTTCAAAGACAGCCTCAC AGTCCTTTTCCGAGCACTCA	175
*LAMB1*	396707	CTTCACCACCTTGGACCACT AGCTGTGGCTCATAGCGAAT	216
*PDPN*	100738269	AGCAGATGCTGTGTCCCTCT TATGGAACCTGGGCTGGTAG	201
*POSTN*	100152401	ATTGACCGTGTCCTCACACA GCCACTTTGTCTCCCATGAT	212
*TAGLN*	397021	TTAAAGGCCGCTGAGGACTA ATGACATGCTTTCCCTCCTG	233
*TMOD1*	100316850	AGCCCTAACGGAAGAAGAGC CCTTTGCTTGCTTTTCCAAG	170
